# FIA- spectrophotometric method for the determination of amoxicillin in pharmaceuticals; application of AES, GAPI, and AGREE greenness assessment tools

**DOI:** 10.1016/j.mex.2023.102437

**Published:** 2023-10-12

**Authors:** Marwa sabbar Falih, Ruba Fahmi Abbas, Neda Ibrahim Mahdi, Nisreen Kais Abood, Mohammed Jasim M Hassan

**Affiliations:** Department of Chemistry, College of Science, Mustansiriyah University, Baghdad, Iraq

**Keywords:** Amoxicillin, Diazotization, Dapsone, FIA, AES, GAPI, and AGREES, *FIA- spectrophotometric method*

## Abstract

A new, simple, and sensitive FIA-spectrophotometric method has been developed for evaluating pure amoxicillin and pharmaceutical formulations. The FIA method involves the reaction of dapsone with sodium nitrite and hydrochloric acid. Subsequently, the diazotized dapsone is coupled with amoxicillin in an alkaline medium, resulting in a stable orange dye with a maximum wavelength of 440 nm. The developed method was validated according to the ICH guidelines and found to have a concentration range of 1–150 µg/mL, a correlation coefficient of 0.9994, a molar extinction coefficient of 0.273 × 10^4^ L/mol.cm, and a detection limit of 0.074 µg/mL. The FIA method was then evaluated using AES, GAPI, and AGREES analytical greenness assessment tools. The FIA method uses dapsone as an eco-friendly reagent, in addition to the FIA method's advantages of reduced sample and reagent usage, reduced waste generation, and cheaper equipment. So, it has been proposed as an excellent eco-friendly method for the determination of AMX in pharmaceutical formulations.

## Graphical abstract

**Figure:**
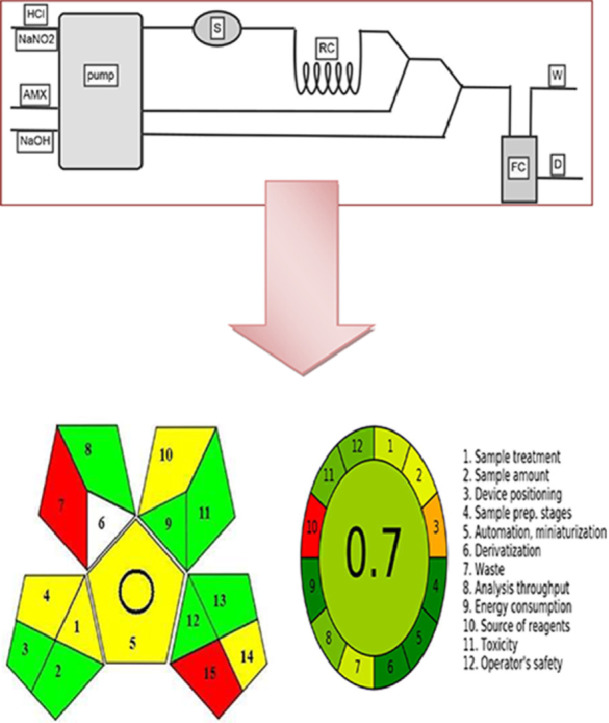

Specifications table

**Table:** 

Subject area:	Chemistry
More specific subject area:	Determination of Amoxicillin
Name of your protocol:	FIA- spectrophotometric method for the determination of amoxicillin in pharmaceuticals; application of AES, GAPI, and AGREE greenness assessment tools
Method name:	FIA- spectrophotometric method
Reagents/tools:	A list of reagents: • Amoxicillin (AMX, 99.99 %) • Dapsone (Dap, 99.99 %) • Sodium hydroxide (NaOH, 99.99 %) • Sodium nitrite (NaNO_2_, 99.99 %) • Hydrochloric acid (HCl, 99.99 %) A list of equipment: • UV–visible spectrophotometer (1800 Shimadzu, Japan) • Digital analytical balance • pH meter (Hana) • Flow injection system
Experimental design:	FIA-spectrophotometric methods have been used for the determination of Amoxicillin in pure and pharmaceutical formulations. The FIA method is greenness and eco-friendliness method by applying greenness assessment tools (AES, GAPI, and AGREE).
Trial registration:	Not applicable
Ethics:	Not applicable
Value of the Protocol:	• The presented data established that FIA methods can be applied for the determination of Amoxicillin with great efficiency. • The FIA method is the eco-friendly method according to the greenness assessment tools (AES, GAPI, and AGREE). • The data is suitable for the determination of Amoxicillin in pure and pharmaceutical formulations and the dataset will also serve as reference material to any researcher in this field.

## Protocol background

Amoxicillin (AMX) is ((2S,5R,6R)−6-[(*R*)-(−)−2-*amino*-2-(*p*-*hydroxyphenyl*)*acetamido*]−3,3-dimethyl-7-oxo-4-thia-1-azabicyclo[3.2.0]heptanes-2-carboxylic acid trihydrate) [1]. It is a penicillin-type semi-synthetic antibiotic in which a thiazolidine ring is attached to a β-lactam ring [2], [3], [4]. The β-lactam group is one of the most widely used antibiotics for the treatment of human and animal infections [5,6]. AMX is used as a first-line antibiotic to treat minor respiratory infections and other common infections [7]. It is better absorbed in vivo than other β-lactam antibiotics and inhibits cell wall synthesis [8]. AMX is manufactured in various forms, including pills, capsules, suspensions, and powders [9]. Its side effects may include nausea, vomiting, rashes, and antibiotic-associated colitis [10].

Dapsone (Dap) is (4,4′-diaminodiphenyl sulfone), which is one of the most important sulfonamides [11], [12], [13]. Dap has a wide range of applications in various skin diseases [14] and is therefore antibacterial and anti-inflammatory [15]. Various analytical methods have been developed to determine AMX, including electrochemistry [16], electrophoresis [17], TLC [18,19], HPLC [20], chemiluminescence methods [21,22], flow [23,24], fluorescence [25], potentiometric titration [26], voltammetric [27,28], and spectrophotometric methods [29], [30], [31]. Most of these methods are highly complex and require toxic organic solvents, special chemicals, and expensive equipment.

Flow injection analysis (FIA) is an automated method of chemical analysis in which the sample is injected into a flowing carrier solution that is mixed with reagents before reaching the detector [32], [33], [34], [35]. This method is characterized by low sample volume (less than 200 µL), reduced reagent consumption (0.5 ml per sampling cycle), short measurement time, simple, cost-effective, less waste, and a high sampling rate (30 to 120 samples per hour). These advantages make Flow Injection Analysis align with the principles of green chemistry [36,37].

The current study aims to employ new estimation methods for AMX in conventional pharmaceuticals. These methods are based on environmentally friendly and cost-effective approaches, are adaptable for use, and provide rapid, automated, reliable, and accurate results without requiring a high level of hands-on experience.

## Experimental

### Instruments

**Fig. 1:**
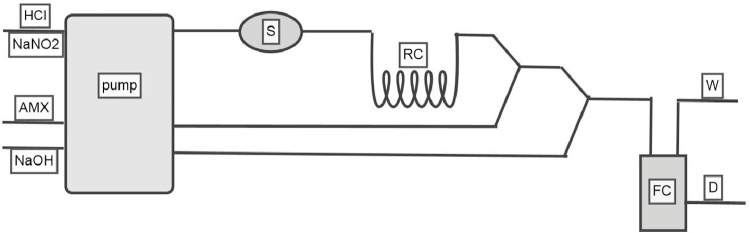
Manifolds adopted for the FIA system include reaction coil(R.C), sample(S), peristaltic pump(P), flow cell(FC), and waste(W).

A digital double-beam spectrophotometer manufactured by Shimadzu in Kyoto, Japan, equipped with quartz cells measuring (1, 0.5) cm, was used to collect the data. As depicted in Fig. 1, local manufacturing was employed to configure a three-channel automated branching flow injection analysis (FIA) system. This manifold includes a multi-channel peristaltic pump (ALITEA, C4, made in Sweden) connected to a 6-port injection valve (Rheodyne, Supelco-USA), which is used for injecting the studied AMX pharmaceutical solutions. The reagents (HCl, NaNO2) and the base (NaOH) solutions were pumped through flexible polyvinyl chloride (PVC) tubing with an inner diameter of 0.8 mm. These solutions then converged with the AMX drug solution and were mixed through a reaction coil made of Teflon tubing (0.8 mm ID).

### Reagent and materials

All the compounds were purchased from Merck and are of analytical purity. The materials for AMX and Dap were generously provided by the State Company for the Drug Industry and Medical Applications (SDI, Iraq). For the batch method, the solutions used were (1000 µg/mL) of AMX and Dap, (25 %) NaOH, (1:1) HCl, and (1 %) NaNO_2_. For the FIA method, the solutions used were (1 mol/L) AMX, (4 × 10–3 mol/L) Dap, (0.6 mol/L) HCl, (1.3 × 10–3 mol/L) NaNO_2_, and 1 M NaOH.

### Preparation of standard solutions

Stock for the batch and FIA techniques, standard AMX and Dap solutions (1000 µg/mL) were made by dissolving 0.1 g of pure AMX and Dap powder into 100 mL of distilled water, (25 %)NaOH, and (1 %) NaNO_2_.

### Standard solutions for pharmaceutical dosage forms

10 capsules of amoxicillin (500 mg from Turkey and India) in a 100 mL volumetric flask, the needed quantity of formulation was dissolved and diluted with distilled water. The resultant solution was filtered through Whatman^Ⓡ^ filter papers to get rid of the insoluble particles.

### General procedure of batch method

Equimolar amounts of 1 mL of (1000 µg/mL) Dap and 1 mL of (1:1)HCl were introduced to a 25 mL graduated flask with the ice bath at (0–5 °C), followed by the slow addition of 1 mL of (1 %)NaNO_2_, waiting 20 min, and then the addition of 1 mL of (1000 µg/mL) AMX and 1.6 mL of (25 %)NaOH. The flask contents were well mixed, diluted with distilled water, and measured at 440 nm with a spectrophotometer using the blank reagent.

### General procedure of fia method

100 µL of (1 mol/L) AMX drug had injected into the carrier stream generated by mixing the three channels. The first channel was used to carry (4 × 10^−^3 mol/L) Dap and the second channel contains carriers of (0.6 mol/L) HCl, and (1.3 × 10^−^3 mol/L) NaNO_2_ using the T-form.

The contribution of NaNO_2_ has in increasing the reaction's speed and completion. This reaction was carried out through the mixing well of a 100 cm long reaction coil. The mixture was passed through an injector and the resulting material reacted with a stream of 1 M NaOH and a flow rate of 3 mL/min in each channel (Fig. 1). Maximum orange solution absorption is calculated to be at 440 nm.

## Results and discussion

### Preliminary study of batch system

**Fig. 2:**
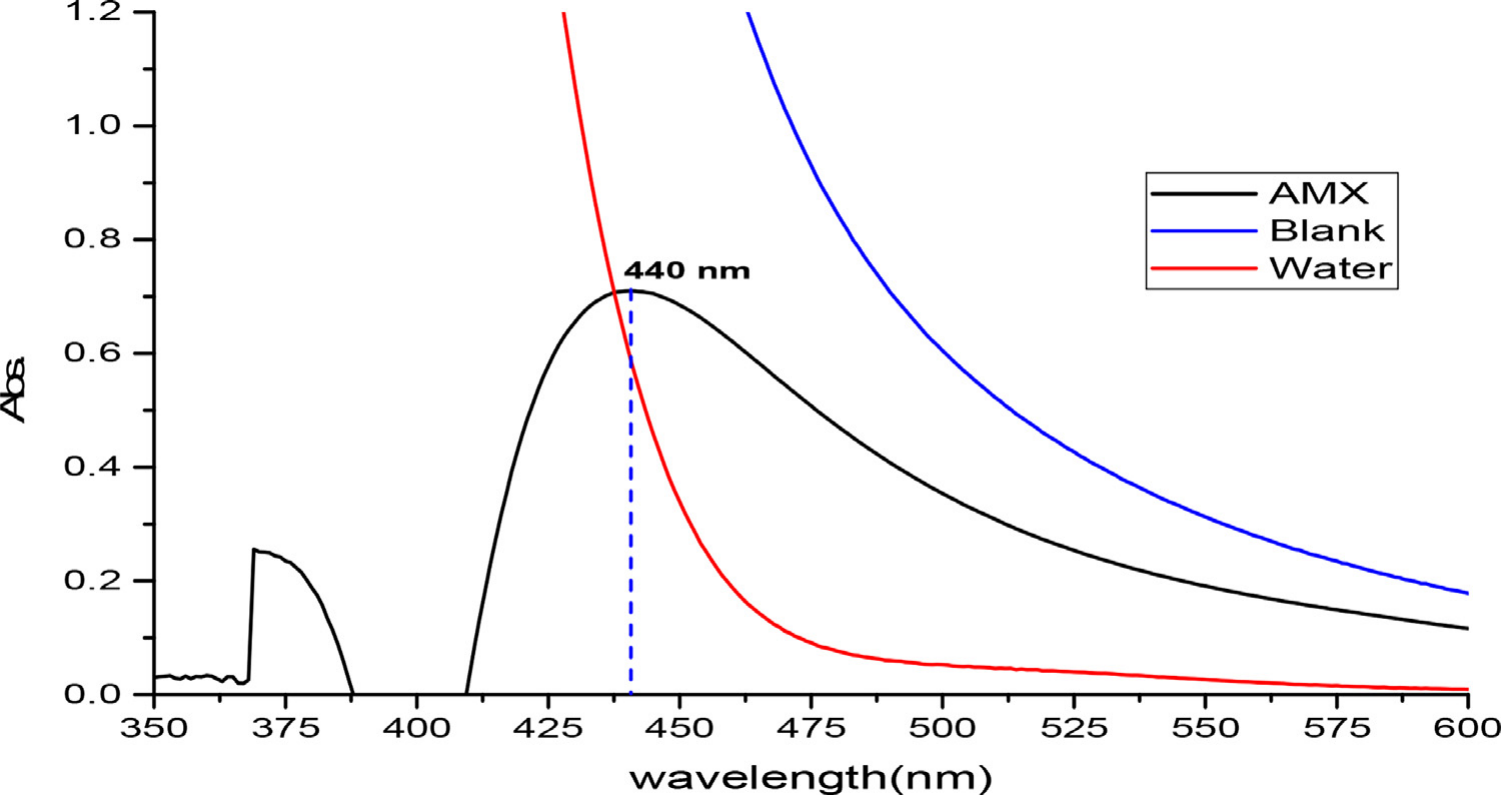
The absorption spectrum of 50 µg/mL AMX against reagent blank (black line), the blank against distilled water(blue line), and AMX product against distilled water (red line) under optimum conditions.

A method of producing an orange color with a 440 nm wavelength by combining diazotized Dap with AMX in an alkaline medium is shown in the Fig. 2.

### Optimum factors of batch method

**Fig. 3:**
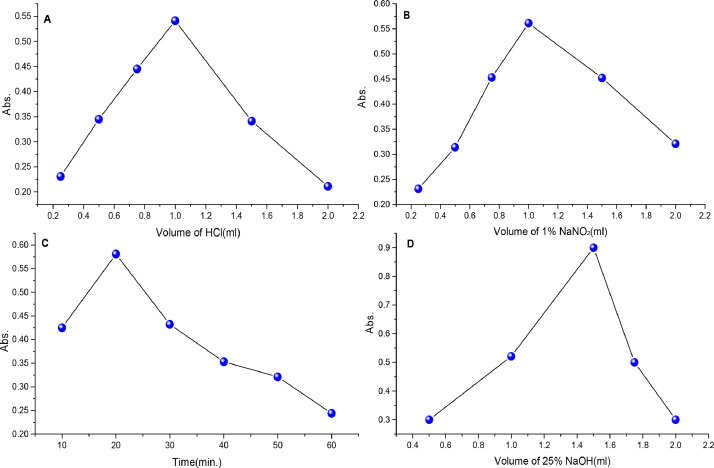
The Effect of experimental conditions of diazotization reaction batch method; (A) volume of acid, (B) volume of NaNO_2_, (C) time, (D) and volume of NaOH.

Specific types of acids and bases are used when preparation of diazonium salts. The role of acid affects the stability and reactivity of the diazonium salt. While, base is used facilitate the formation of the diazonium salt. In this study, many acids and bases were tried such as (HCl, H_2_SO_4_, CH_3_COOH, HNO_3_, KOH, NaOH, Na_2_CO_3_, and NH_4_OH), HCl and NaOH were selected because they exhibited the highest absorption during the diazotization process. The absorption of orange dyes has been shown to be affected by many factors. Different volumes (0.25 to 2 mL) of HCl were studied and 1 mL of HCl was used to achieve the highest absorbance(Fig. 3A). The volume of 0.144 M (1% w/v) NaNO_2_ was determined from 0.25 to 2 mL, the maximum absorbance occurred at a volume of 1 mL(Fig. 3B). Subsequently, The time required to complete the reaction after the addition of NaNO_2_ is 20 min(Fig. 3C). To ascertain the ideal concentration of NaOH, we also examined different quantities of 6.25 M NaOH from 0.5 to 2 mL, the maximum absorption was achieved using 1.5 mL of 6.25 M NaOH(Fig. 3D).

**Scheme 1:**
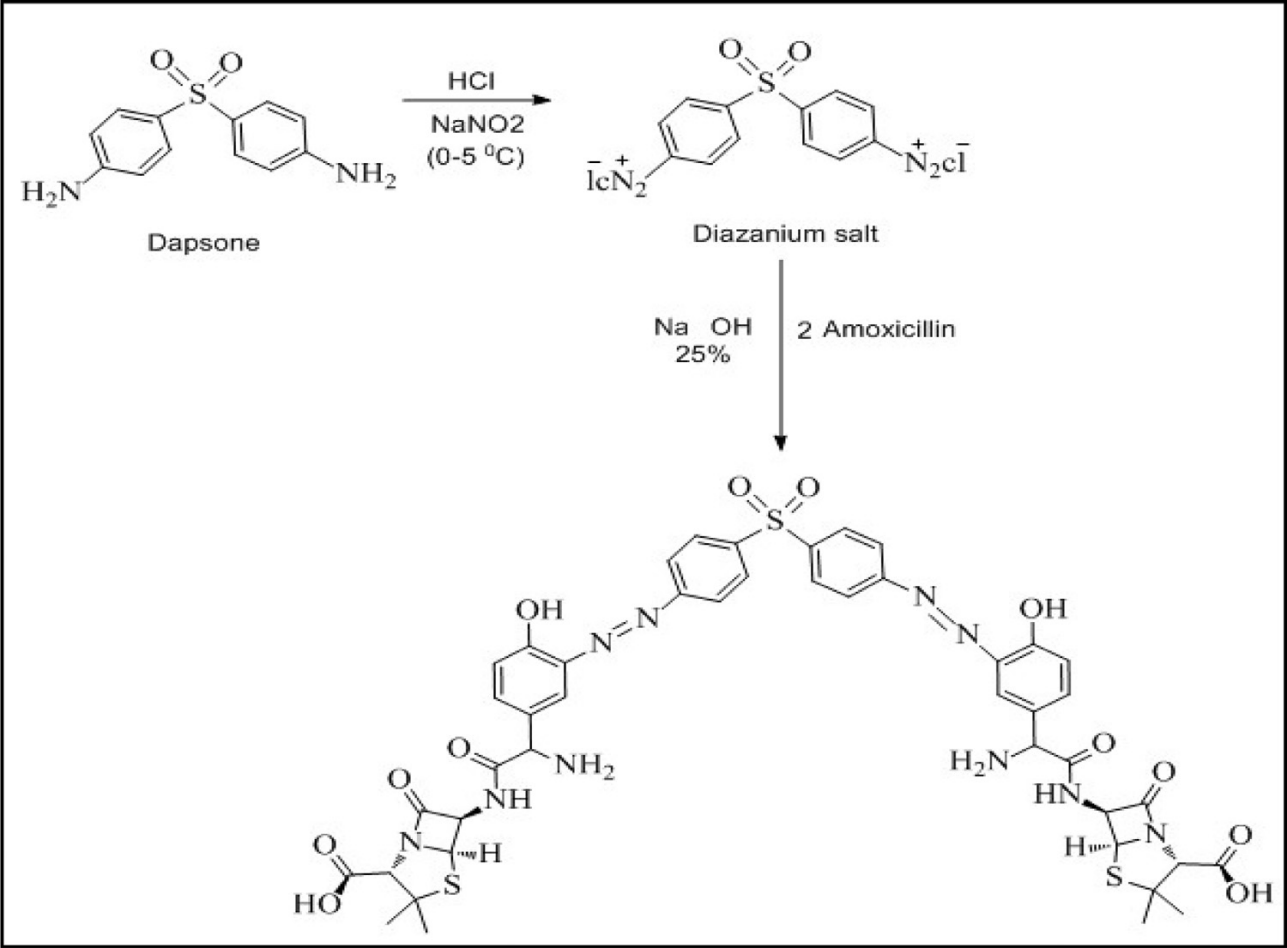
The suggested mechanism reaction of Dap and AMX to form azo dye.

The suggested mechanism of the amino pharmaceutical of Dap reacts with nitrous acid to form a diazonium salt, which combines with AMX in alkaline media to produce the azo dye as in Scheme 1.

### Optimum factors of FIA method

**Fig. 4:**
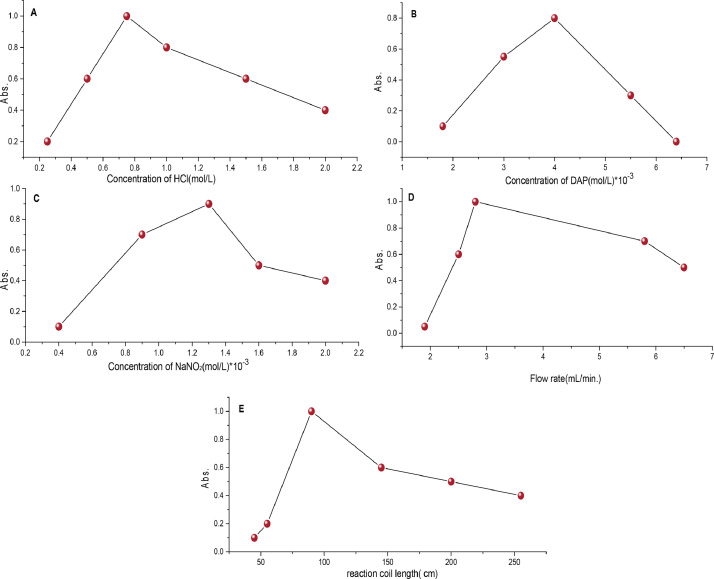
The Effect of experimental conditions of FIA method; (A) concentration of HCl, (B) concentration of DAP, (C) concentration of NaNO_2_, (D) flow rate, and (E) reaction coil length.

Flow injection analysis (FIA) is an automated method in which a sample analyte is injected into a continuous flow of a carrier solution, which mixes with other reagents before reaching a UV–Visible spectrophotometer detector. The influence of various experimental parameters on the FIA method, including the different Concentration of HCl, Dap, and NaNO_2_ solutions (Fig. 4A–C).The effect of flow rate on the sensitivity of colored solution was studied in the range of 1–5 mL/min. It seen that the absorbance increased when flow rate of 3 mL/min (Fig. 4D). Reaction coil length is an important parameter that affects sensitivity. The coil length was investigated from 25 to 250 cm. The results obtained showed that a coil length of 100 cm gave the highest absorbance as shown in (Fig. 4E). Beyond this length, the absorbance of the colored solution decreases as the length of the coil increases due to increased dilution due to dispersion. The results of the values of the experimental conditions for FIA are shown in Table 1.

**Table 1:** The optimal conditions of FIA method.

Variable	Range of study	Optimum value
Concentration of HCl(mol/L)	0.2–2	0.7
Concentration of Dap(mol/L)	1. 5 × 10^−3^–6.5 × 10^−3^	4 × 10^−3^
Concentration of (1 %)NaNO_2_(mol/L)	0. 4 × 10^−3^–2 × 10^−3^	1.3 × 10^−3^
Flow rate(mL/min)	1- 5	3
Reaction coil length(cm)	25–250	100

### Analytical characteristics

**Fig. 5:**
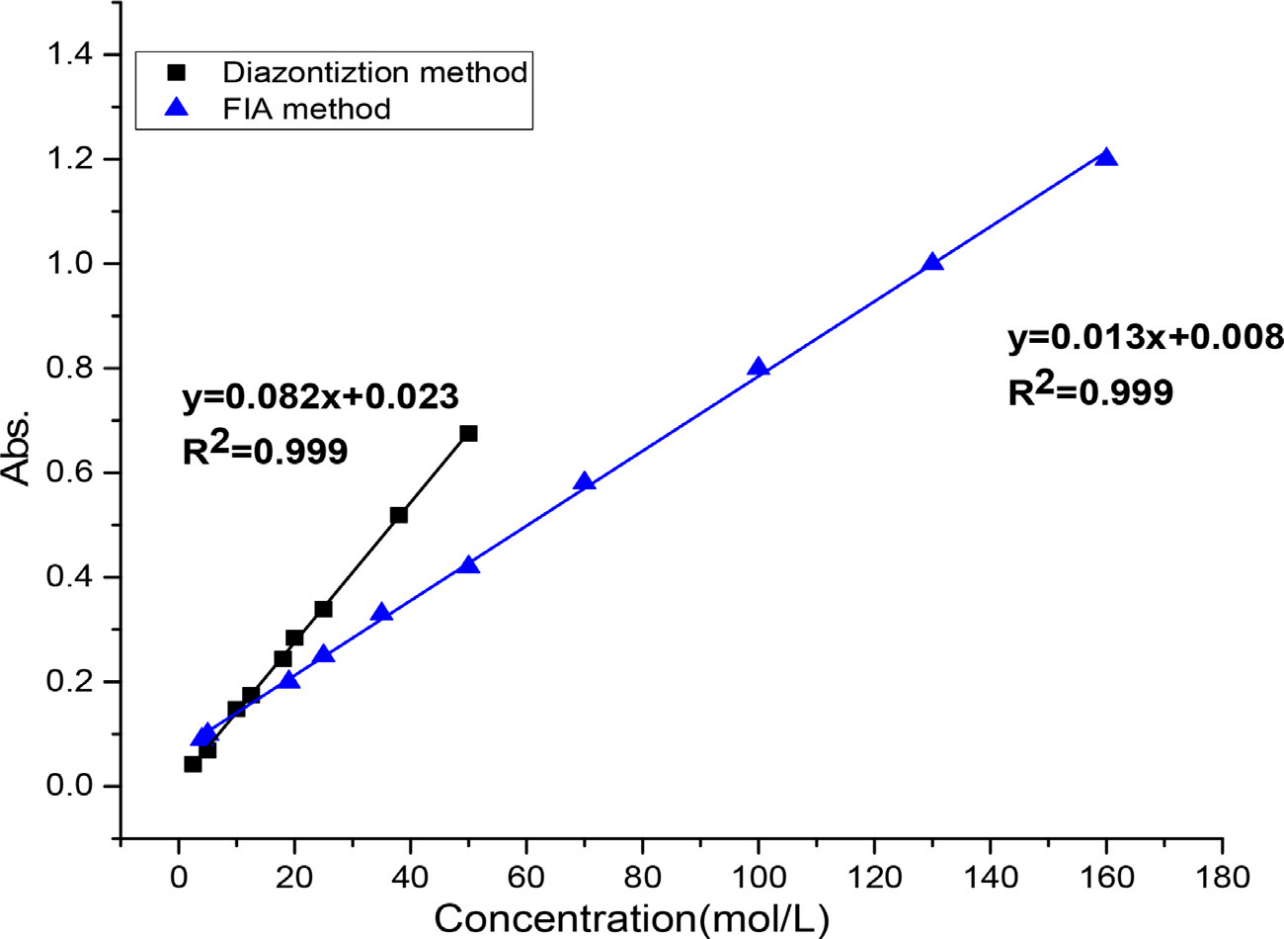
Calibration curves of suggested methods.

After optimization of experimental conditions, two calibration curves for the suggested methods were prepared by plotting the absorbance vs. concentrations of AMX. The small values of Sandell's sensitivity, slope, and intercept parameters in the calibration curves provide evidence that the diazotization and FIA methods are precise, accurate, and reliable for the analysis conducted in the study (Fig. 5 and Table 2).

**Table 2:** Regression and analytical parameters for the analysis of AMX using batch and FIA methods.

Parameters	batch	FIA
λ _max_ nm	440	440
Color	Orange	Orange
Regression equation	*Y* = 0.082x+0.023	*Y* = 0.013x-0.008
Linearity range(µg/mL)	(2.5 − 50)	(1–150)
Correlation Coefficient (r)	0.9994	0.9994
Ɛ(L/mol.cm)	0.98 × 10^4^	0.273 × 10^4^
Sandal’ sensitivity (µg/ cm^2^)	0.063	0.08
Slope (b)	0.082	0.013
Intercept(a)	0.023	- 0.008
Limit of detection LOD(µg/mL)	0.194	0.074
Limit quantification LOQ(µg/mL)	0.540	0.375

LOD = 3.3 x SD_b_/b, LOQ= 10 x SD_b_/b, SDa = Standard deviation of blank.

### Accuracy and precision

The accuracy and precision of batch and flow injection methods were investigated by measuring the absorbance with at least 5 readings for each concentration under optimal conditions using different concentrations. Precision and accuracy are determined by RE (%), R (%), and RSD (%) as shown in Table 3.

**Table 3:** Accuracy and precision for the proposed methods of pure AMX.

Drug	Method	Amount of drugs (µg /mL)		E%=$\frac{CF-CT}{CT}$*100	Rec%= E%+100	Average Rec%	RSD%= $\frac{SD}{mean}$*100 (*n* = 5)
		C_T_	C_F_				
AMX	batch	2.5	2.54	3.5	103.5	101.3	4.5
		18	17.91	0.5	100.2		0.65
		50	49.89	0.22	100.22		0.28
	FIA	20	20.1	0.5	100.5	100.06	0.42
		60	59.87	−0.22	99.78		0.15
		70	69.43	−0.11	99.89		0.13

C_T_ and C_F_ are taken and found concentration of AMX, E% is relative error, Rec%= recovery, and RSD is relative standard deviation.

**Application:** batch and flow injection methods had been applied for the analysis of AMX in commercial capsules (500 mg per tablet) from Turkey and India. The results in Table 4 show a good agreement with label claims.

**Table 4:** Analysis of AMX in commercial capsule samples.

Drug	Type of AMX commercial capsules (500 mg per tablet)	Method	Amount of drugs (µg /ml)		E%=$\frac{CF-CT}{CT}$*100	Rec%=*E*%+100	Average Rec%	RSD%=$\frac{SD}{mean}$*100 (*n* = 5)
			C_T_	C_F_				
AMX	AMX capsules Turkey	Batch	10.00	10.33	3.30	103.30	101.1	0.13
			25.00	25.04	0.16	100.16		0.03
			38.00	37.94	−0.16	99.84		0.25
	AMX capsules Indian		10.00	10.26	2.60	102.60	100.79	0.95
			25.00	24.96	−0.16	99.84		0.38
			38.00	37.97	−0.08	99.92		0.15
	AMX capsules Turkey	FIA	20	19.86	−0.7	99.3	99.96	0.56
			25	25.05	0.2	100.2		0.42
			40	40.15	0.38	100.38		0.26
	AMX capsules Indian		20	19.85	−0.75	99.25	98.92	0.42
			30	29.53	−1.57	98.43		0.28
			40	39.63	−0.93	99.08		0.2

C_T_ and C_F_ are taken and found concentration of AMX, E% is relative error, Rec%= recovery, and RSD is relative standard deviation.

### Greenness evaluation

In the last few years, most analytical chemistry studies focused on the greenness assessment to adopt eco-friendly and sustainable analytical methods. The aim of this work is to use the FIA method as an eco-friendly method by applying greenness assessment tools (AES, GAPI, and AGREE). First, an Analytical Eco-Scale (AES) was utilized for evaluating the FIA method; The FIA method achieved a score of 86, indicating its excellence as a green methodology. The high score of the FIA method is due to the amount of sample and waste of less than 2 mL, Dapsone (used as a reagent) does not fall under the hazardous according to the EPA's Toxic Release Inventory nor is it classified as PBT (Persistent, Bioaccumulative, and Toxic), and energy of flow system with UV detector ≤ 0.1 kWh per sample [38].

Second, the Green Analytical Procedure Index (GAPI) is a green analytical evaluation tool that has been used for investigating the greenness of the FIA method used in the determination of AMX. Increasing the green color in the pentagram shape of GAPI means a safe method, the yellow color medium hazard, while red refers to a hazard or non-green method [39], [40], [41]. The FIA is a greener method due to it being a direct and fast method, with no extraction steps, and the small volume solutions used (that leads to obtaining less waste).

Third, the greenness of the FIA method was investigated using the AGREE (Analytical Greenness Metric Approach) software by evaluating 12 parameters of green analytical aspects. It is calculated using software that operates on 12 parameters, corresponding to the 12 green analytical chemistry (GAC) principles. The final score of AGREE is a fraction of unity, from 0 to 1. The theory of 12 GAC principles appears as a classic clock shape with numbers 1–12 on the circle's side in the AGREE tool. When the value closest to 1 indicates that the technique is ecologically friendly. In this study, the FIA method score was found to be 0.7 indicating the greenness of the method (Table 5) [42,43].

**Table 5:** AES, GAPI, and AGREE parameters for the description of FIA methods used for the determination of AMX.

method	Analytical Eco-Scale (AES)		Penalty points (pp)
FIA method	Step 1: amount	Less than2 mL	1
	Step 2: chemicals	HCl	4
		NaOH	2
		NaNO2	4
		Dapsone	0
	Step 3: energy	flow system with UV detector (≤ 0.1 kWh per sample)	0
	Step 4: waste	Less than2 mL	3
		Total pp	14
		**Eco-Scale**	100–14=86
	**GAPI parameters**		**GAPI Assessment**
	1-Collection(on-line or at-line)_, 2-_Preservation(none), 3-Transport(none), 4-Storage(under normal condition, room temperature), 5- General method type (simple procedure). **Sample preparation**: 6-Scale of extraction (none), 7-Solvents and reagents(non-green solvents/reagents used), 8-Additional treatments (none). **Reagent and solvents**: 9-Reagents and solvents amounts(<10 mL), 10- NFPA health hazard- moderate toxic NFPA 2 or 3 (NFPA health hazard of NaOH, and HCl rating are 3, NFPA health hazard of NaNO_2_ score is 2, and Dap score is 0), 11-NFPA safety hazard(the flammability score of all reagents is 0) **Instrumental (flow system with UV detector):** 12- ^(^<0.1 kWh per sample), 13-Occupational hazard (Hermetic sealing of the analytical process), 14-Waste(1–10 ml), 15- Waste treatment (no treatment)		
	**AGREE parameters**		**AGREE Assessment**
	1- at-line analysis, 2-amount of sample (less than 2 mL), 3- location of the analytical device (at-line), 4- number of steps of chemical analysis(3 or fewer), 5- degree of automation (automatic) and sample preparation (miniaturized), 6- none derivatization agents used, 7-waste amount (less than 2 mL), 8- number of samples are determined (30 samples per hour), 9-energy of flow system with UV detector (<0.1 kWh per sample), 10-none of reagents are bio-based source, 11- toxic reagents (yes), 12- threats (toxic to aquatic life)		

### Comparison with literature methods

Tables 6 and 7 portrays a brief comparison of the flow injection method with some previously published methods. It can be observed that the developed FIA method scored a higher greenness value than the other methods. Moreover, LOD, LOQ, and Linearity obtained are close to the results obtained from previously published methods, which indicates that the FIA method has their suitability for use in AMX drug identification.

**Table 6:** Comparison of the three proposed method with some recent studies on determination of AMX.

Method	Reagents	AES	GAPI	λ_max_ nm	Ref
CPE based on diazotization reaction- spectrophotometric	• Metoclopramide hydrochloride • Na_2_CO_3_ • Triton X-114 • ethanol • HCl • NaNO2 • NaOH • KOH • NH4OH	Amount 2 + chemicals 31+energy 0+ waste 3 = 38 **Eco-scale**=64			44
Flow injection based on diazotization reaction - spectrophotometric method-	• p-Nitroaniline • HCl • NaNO2 • NaOH • ethanol	Amount 2 + chemicals 20+energy 0+ waste 3 = 25 **Eco-scale**=75			45
HPLC–ESI mass spectrometry	• Acetonitrile • dichloromethane • formic acid, • acetic acid • trichloroacetic acid • perchloric acid • ammonium acetate	Amount 3 + chemicals 33+energy 1+ waste 5 = 42 **Eco-scale**=58			45
FIA- based on diazotization reaction - spectrophotometric method	• Dapsone • HCl • NaNO2 • NaOH	Amount 1 + chemicals 10+energy 0+ waste 3 = 14 **Eco-scale**=86			This study

**Table 7:** Comparison of the FIA method with some recent studies on determination of AMX.

Method	LOD (µg/ ml)	LOQ (µg/ ml)	Linearity (µg/ ml)	λ_max_	Ref
CPE based on diazotization reaction- spectrophotometric	0.083	0.29	0.3–3.0	479	44
Flow injection based on diazotization reaction - spectrophotometric method-	0.104	—-	0.5–100.0	478	45
HPLC–ESI mass spectrometry	0.04	0.12	0.125–8	——	46
FIA- based on diazotization reaction - spectrophotometric method	0.074	0.375	1–150	440	This study

## Conclusions

A quick, sensitive, and environmentally friendly approach for determining AMX in pure samples and pharmaceuticals was developed. The FIA-diazotization process is based on the use of dapsone (which does not fall under hazardous materials) as the green reagent, rather than a costly and poisonous alternative. This technique is entirely eco-friendly and sustainable due to its high sample throughput, low reagent consumption, and the use of ecologically benign dapsone. For the FIA method, in accordance with ICH standards, an RSD of less than 0.95 % indicates the method's reproducibility, reliability, and limitations. The FIA technique was employed to automate the estimation of AMX in medicines. The described methods have been effectively applied for the assay of AMX in pharmaceutical capsules with respectable accuracy.

## CRediT authorship contribution statement

**Marwa sabbar Falih:** Validation, Investigation. **Ruba Fahmi Abbas:** Project administration, Writing – original draft, Writing – review & editing. **Neda Ibrahim Mahdi:** Methodology. **Nisreen Kais Abood:** Funding acquisition, Project administration. **Mohammed Jasim M Hassan:** Formal analysis.

## Declaration of Competing Interest

The authors declare that they have no known competing financial interests or personal relationships that could have appeared to influence the work reported in this paper

## Data Availability

Data will be made available on request.
